# Mechanistic Insights and Anti-cancer Activity of 2H-benzo[b][1,4]oxazin-3(4H)-one Linked 1,2,3-Triazoles in Human Cancer Cells

**DOI:** 10.3389/fphar.2025.1565657

**Published:** 2025-06-13

**Authors:** Qingying Liu, Xixi Hou, Mingyue Tian, Baoyu He, Jingjing Guo, Yajie Guo, Jianxue Yang

**Affiliations:** ^1^ The First Affiliated Hospital , and College of Clinical Medicine of Henan University of Science and Technology, Luoyang, Henan, China; ^2^ Department of Pain Medicine, The First Affiliated Hospital of Zhengzhou University, Zhengzhou, Henan, China; ^3^ Centre for Artificial Intelligence Driven Drug Discovery, Faculty of Applied Sciences, Macao Polytechnic University, Macao, China; ^4^ Department of Emergency, The Eighth Affiliated Hospital, Sun Yat-Sen University, Shenzhen, China

**Keywords:** anticancer, apoptosis, 2H-benzo[b][1,4]oxazin-3(4H)-one, 1, 2, 3-triazole, ROS, DNA damage

## Abstract

A series of 2H-benzo[b][1,4] oxazin-3(4H)-one derivatives linked to 1,2,3-triazoles were designed, synthesized, and evaluated for their anticancer activity in several human cancer cell lines, including A549 (lung), Huh7 (liver), MCF-7 (breast), HCT-116 (colon), and SKOV3 (ovary). Cell viability assays revealed that these compounds exhibited the most potent activity against A549 cells. Among them, compounds 14b and 14c demonstrated the strongest inhibitory effects, with IC_50_ values of 7.59 ± 0.31 μM and 18.52 ± 0.59 μM, respectively. Flow cytometry analysis further confirmed that compounds 14b and 14c induced significant apoptosis. Additional studies showed that these compounds elevated reactive oxygen species (ROS) levels, which may contribute to apoptosis. Moreover, compounds 14b and 14c notably increased the number of dead cells while reducing viable cell counts. Western blot analysis indicated that these compounds could induce DNA damage and autophagy, which may play a key role in their anticancer effects.

## 1 Introduction

According to estimates from the World Health Organization (WHO), approximately 14 million new cases of cancer are diagnosed worldwide each year, and this number continues to rise. By 2030, the annual incidence is projected to reach 21 million. Cancer remains a leading cause of death in both developing and developed countries, with nearly 10 million fatalities reported annually. However, conventional anticancer drugs often exhibit limited efficacy and severe toxic side effects, underscoring the urgent need for the development of novel therapeutic agents. A key strategy in anticancer drug design is molecular hybridization, which involves the combination of pharmacologically active molecular fragments to enhance therapeutic effectiveness while minimizing toxicity (Fiste et al., 2024).

**FIGURE 1:**
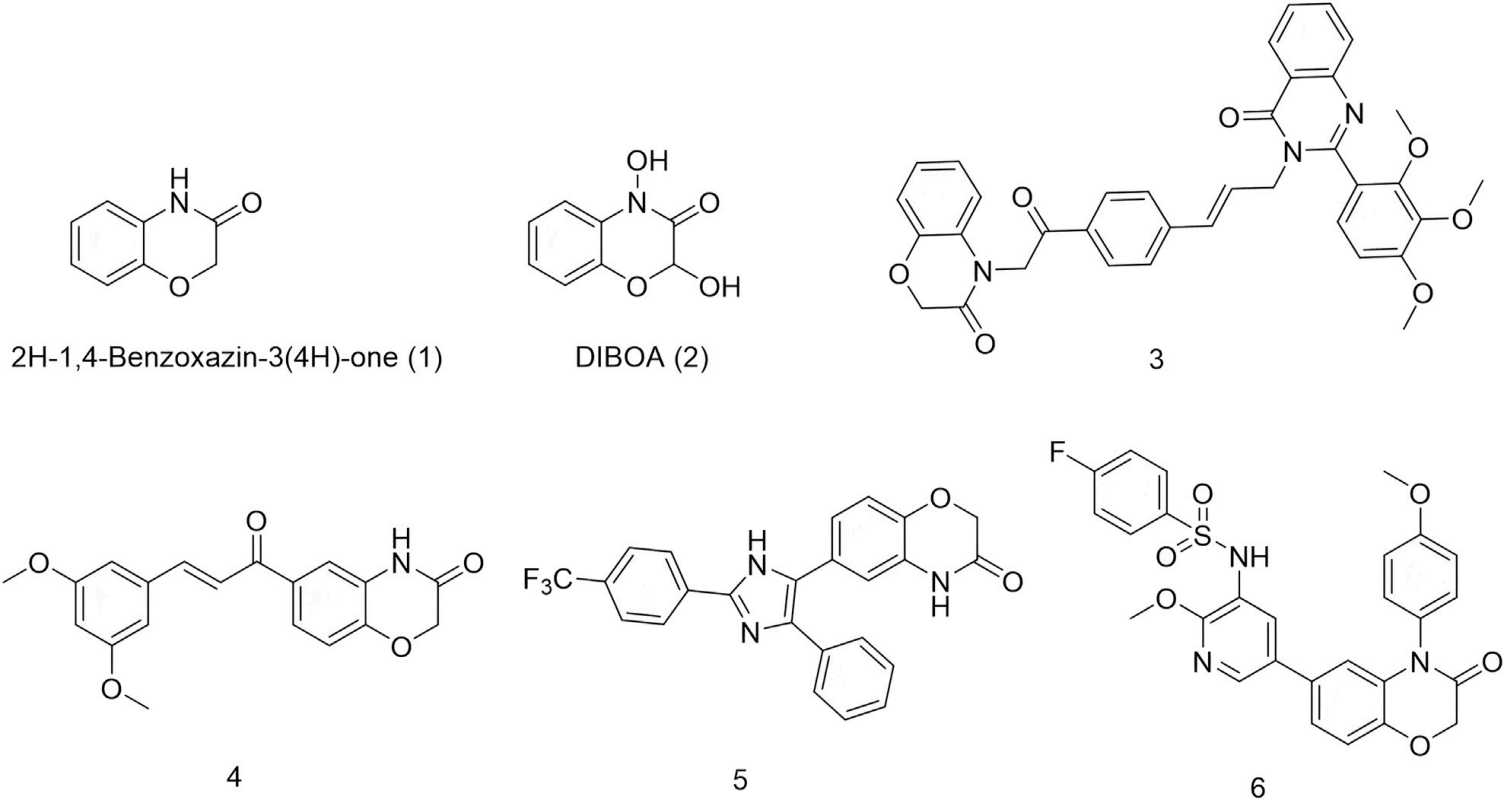
Structures of synthesized 2H-1,4-benzoxazinone derivatives.

Natural products are a crucial source for new drug discovery as their complex and unique chemical structures confer a broad spectrum of biological activities, making them valuable lead compounds. One plant secondary metabolite is 2H-1,4-Benzoxazin-3(4H)-one (1), which plays a key role in plant defense mechanisms Figure 1. Its derivatives are primarily found in wheat and other grasses (Hashimoto et al., 1991; Hasui et al., 2013; Zamani et al., 2021). For example, DIBOA (2), a benzoxazinone derivative found in oats, contains an electrophilic lactam moiety that reacts with the C-8 carbon atom of guanine, leading to DNA mutations (Campos et al., 1988; Mhmood et al., 2019). This mechanism endows plants with insecticidal and antifungal properties (Shudo et al., 1983). Due to its DNA interaction capability and relatively low biological toxicity, numerous benzoxazinone-based derivatives have been designed and synthesized for anticancer drug research (Kwiecien et al., 2016; Armitage et al., 2012). For instance, Nagarapu and colleagues synthesized a series of 1,4-benzoxazinone-acetylphenylallyl quinazolin-4(3H)-one hybrids that exhibited inhibitory effects against A549, HeLa, and MDA-MB-231 cancer cells. Among them, compound 3 demonstrated remarkable potency against A549 cells, with a GI_50_ value of 0.32 μM (Bollu et al., 2017). Similarly, Zhou et al. (2014) developed a series of 6-cinnamoyl-2H-benzo[b][1,4]oxazin-3(4H)-one derivatives, among which compound 4 effectively suppressed A549 lung cancer cell growth by inducing autophagy and cell cycle arrest. Additionally, Manojit’s research group synthesized compound 5, a 2H-1,4-benzoxazin-3(4H)-one derivative, which significantly reduced the viability of A549, DLD-1, and MV4-11 cells, demonstrating superior efficacy compared to erlotinib (Rajitha et al., 2011). Li and colleagues designed compound 6, a PI3K/mTOR inhibitor based on the 1,4-benzoxazinone scaffold. This compound exhibited potent activity against PI3Kα, with an IC_50_ value of 0.63 nM, and demonstrated good oral bioavailability in mice. It also showed significant inhibitory effects on HeLa and A549 cells, with IC_50_ values of 1.35 μM and 1.22 μM, respectively (Yan et al., 2019).

1,2,3-Triazole is a crucial pharmacophore in drug design (Weide et al., 2010; Guan et al., 2024). Its electronic isosterism with functional groups such as amides, esters, and carboxylic acids allows coordination with metal ions, enabling interactions with metalloproteases and inhibiting their activity (Ye et al., 2014; Hou et al., 2024), thereby influencing tumor cell growth (Ying-Chao et al., 2013; Hou et al., 2023; Zhou et al., 2023). Additionally, its planar and rigid structure facilitates intercalation into tumor cell DNA, leading to interactions that induce DNA damage. These properties have made 1,2,3-triazole-based compounds widely utilized in anticancer drug development. For instance, Xie et al. (2023) synthesized cabotegravir derivatives by incorporating various 1,2,3-triazole substituents into the structure of the anti-HIV drug cabotegravir. Cytotoxicity assays against HepG2 cells revealed significant inhibitory effects in the majority of derivatives, with compound 7 demonstrating potent activity (IC_50_ = 4.07 ± 0.09 μM). Further studies showed that compound 7 induced apoptosis, G2/M phase arrest, and DNA damage while inhibiting cell invasion and migration in a concentration-dependent manner. Mechanistic investigations revealed that compound 7 triggered apoptosis via the mitochondrial pathway by increasing the Bax/Bcl-2 ratio and activating cleaved caspase-9, cleaved caspase-3, and cleaved PARP (Xie et al., 2023). Similarly, Mohammed’s research group designed and synthesized a series of novel 1,2,3-triazole-linked ciprofloxacin-chalcones that exhibited remarkable antiproliferative activity against colorectal cancer cells. Among them, compound 8 demonstrated potent inhibitory activity against HCT116 cells (IC_50_ = 4.87 ± 0.78 μM) and significantly inhibited topoisomerase I, topoisomerase II, and tubulin polymerization. It also upregulated γ-H2AX protein expression, indicating its ability to induce DNA damage. Furthermore, compound 8 arrested HCT116 cells in the G2/M phase via the ATR/CHK1/Cdc25C signaling pathway (El Hamaky et al., 2024). Nukala’s research team synthesized clioquinol-linked annulated 1,2,3-triazole hybrids and evaluated their *in vitro* anticancer activity against four human cancer cell lines (MCF-7, HeLa, A549, and PC33). Screening results revealed varying degrees of inhibition, with compound 9 exhibiting the highest potency against HeLa cells (IC50 = 3.38 ± 1.14 μM), comparable to the standard anticancer drug etoposide. Molecular docking studies further demonstrated that compound 9 strongly binds to topoisomerase II (Srinivas Reddy et al., 2022). Nasr’s research group designed a series of quinazoline-1,2,3-triazole hybrids that were evaluated for their anticancer potential. MTT assay results demonstrated that compound 10 exhibited significant inhibitory activity against HCT116 cells (IC_50_ = 10.63 ± 0.80 μM) and notably inhibited topoisomerase II activity (IC_50_ = 19.74 ± 0.79 μM) (Mohammed et al., 2022) Figure 2.

**FIGURE 2:**
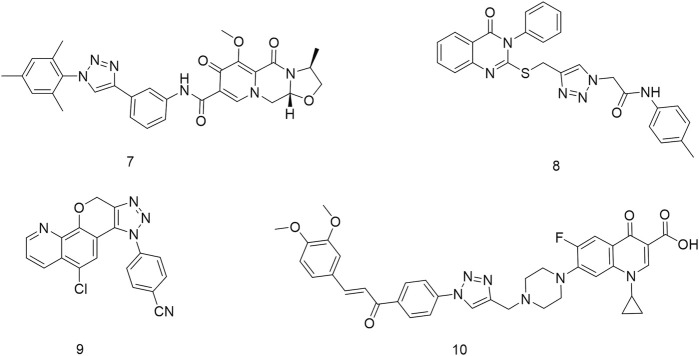
Structures of 1, 2, 3-triazoles with antitumor activity.

Structural hybridization involves combining two or more molecules with similar or complementary pharmacological properties into a single hybrid entity through methods such as coupling or chemical bond formation. This approach generates a new molecule that retains the advantageous properties of both components, potentially enhancing efficacy, improving drug absorption, reducing toxicity, increasing solubility, and extending the drug’s half-life, ultimately leading to improved therapeutic outcomes. As a result, structural hybridization has become a key strategy in drug development. Given the promising anti-tumor activity of 2H-1,4-benzoxazin-3(4H)-one and the widespread use of the 1,2,3-triazole scaffold in anti-tumor drug design, we aimed to modify 2H-1,4-benzoxazin-3(4H)-one by incorporating the 1,2,3-triazole moiety through structural hybridization. The resulting compounds will then be evaluated for their anticancer potential.

## 2 Chemistry

**SCHEME 1:**
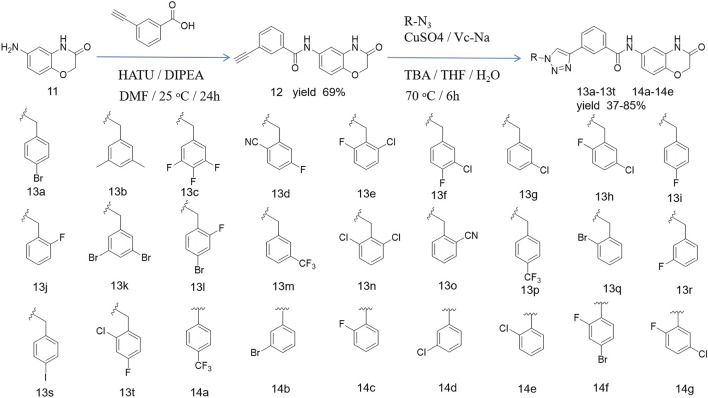
Reaction routes to compounds 13a–13t and 14a–14 g.

This reaction route was consistent with our previous work (Hou et al., 2024). In this route, 6-amino-2H-benzo[b][1,4]oxazin-3(4H)-one (11) was used as a raw material, and it was condensed with 3-ethynylbenzoic acid under the action of HATU and DIPEA to obtain the terminal alkyne compound 12. Compound 12 was reacted with azide compounds having different substituents to obtain target compounds (**13a–13t** and **14a–14g**) (Scheme 1). The structures of the target compounds were confirmed through ^1^H and ^13^C nuclear magnetic resonance (^1^H NMR and ^13^C NMR) spectroscopy.

## 3 Results and discussion

### 3.1 Cell viability of novel synthetic compounds

In order to investigate the anticancer activity of new synthetic 1,4-benzoxazinone-linked 1,2,3-triazole compounds, we chose several cancer cell lines, including A549 (lung), Huh7 (liver), MCF-7 (breast), HCT-116 (colon), and SKOV3 (ovary), and measured cell viability using the CCK-8 assay. Cells were exposed to 40 μM of the compounds for 48 h, and then cell viability was determined. The results are shown in Table 2. Data revealed that the new compounds exhibited more effective activity on A549 cell lines than others. Among those compounds, **13e**, **13f**, **13h**, **13q**, **14b**, **14c**, **14d, 14e, 14f, and 14g** had an inhibition rate higher than 50%. In addition, we used the reference drug gefitinib as a positive control and measured its cell viability on different cell lines. The results showed that gefitinib had effective activity on A549 cells. We used BEAS-2B cells as a normal cell line to determine the toxic effects of these compounds on normal cells. The results (Table 1) indicated that these novel compounds had little or no toxic effect on normal cells. Furthermore, we evaluated the IC_50_ of these compounds in A549. The data (Table 2) suggests that compounds **14b** and **14c** had more remarkable anticancer activity with IC_50_ values of 7.59 ± 0.31 μM and 18.52 ± 0.59 μM, respectively.

**TABLE 1:** Cell viability of new compounds.

No.	Cell viability (100%), 40 μM, 48 h					
	A549	Huh7	MCF-7	HCT-116	SKOV3	BEAS-2B
**13a**	104.83 ± 3.60	109.14 ± 2.48	107.32 ± 3.39	114.71 ± 4.01	104.28 ± 2.08	109.97 ± 1.16
**13b**	114.51 ± 1.06	107.73 ± 1.90	112.39 ± 2.33	124.35 ± 4.48	114.71 ± 2.08	105.74 ± 0.38
**13c**	62.95 ± 1.00	109.97 ± 1.20	91.38 ± 2.78	68.19 ± 3.35	88.55 ± 1.16	100.88 ± 1.59
**13d**	132.87 ± 1.67	126.35 ± 1.71	136.74 ± 1.49	135.23 ± 3.97	119.72 ± 1.47	96.56 ± 2.60
**13e**	45.95 ± 0.35	126.11 ± 1.82	119.56 ± 3.06	60.88 ± 1.95	85.83 ± 4.65	108.12 ± 1.19
**13f**	44.51 ± 3.23	88.43 ± 1.30	85.49 ± 2.20	68.05 ± 5.40	83.67 ± 1.74	123.57 ± 3.82
**13g**	99.48 ± 3.00	103.61 ± 3.80	104.52 ± 0.28	89.77 ± 1.75	109.37 ± 2.06	103.09 ± 0.72
**13h**	44.11 ± 2.78	105.12 ± 2.37	94.17 ± 2.87	80.39 ± 3.0	89.66 ± 5.20	124.89 ± 1.84
**13i**	129.96 ± 1.04	119.63 ± 2.62	124.59 ± 0.87	142.58 ± 0.97	124.22 ± 1.08	116.59 ± 2.34
**13j**	53.64 ± 2.43	97.22 ± 2.59	106.81 ± 2.73	60.33 ± 1.30	92.73 ± 0.81	125.77 ± 0.46
**13k**	140.65 ± 0.11	128.99 ± 2.00	140.53 ± 1.94	151.71 ± 3.50	134.93 ± 2.40	112.44 ± 1.63
**13l**	57.02 ± 2.02	119.18 ± 2.06	105.14 ± 2.42	109.88 ± 5.15	91.62 ± 2.21	101.50 ± 0.49
**13m**	126.25 ± 1.90	124.28 ± 3.41	126.92 ± 1.71	144.27 ± 2.31	126.22 ± 2.44	107.94 ± 0.77
**13n**	55.65 ± 1.02	129.38 ± 0.81	121.95 ± 3.23	83.12 ± 1.96	89.32 ± 1.41	104.68 ± 0.69
**13o**	64.05 ± 6.89	103.75 ± 2.70	103.49 ± 2.66	97.90 ± 1.02	98.37 ± 2.58	108.83 ± 1.51
**13p**	94.65 ± 6.71	119.22 ± 1.70	113.48 ± 1.67	120.31 ± 2.54	104.33 ± 1.06	102.12 ± 4.05
**13q**	29.34 ± 1.12	122.66 ± 1.89	97.91 ± 2.13	77.09 ± 2.31	89.51 ± 3.23	114.92 ± 0.85
**13r**	69.97 ± 2.73	130.90 ± 2.34	122.34 ± 2.48	74.87 ± 3.73	115.17 ± 0.67	96.29 ± 1.01
**13s**	128.77 ± 1.19	137.18 ± 1.61	127.23 ± 3.34	150.61 ± 1.11	117.75 ± 1.64	94.67 ± 1.56
**13t**	96.60 ± 2.07	101.33 ± 3.53	97.86 ± 2.53	113.82 ± 1.76	97.17 ± 1.54	103.17 ± 2.67
**14a**	88.38 ± 7.15	113.61 ± 1.46	100.65 ± 3.09	109.79 ± 5.19	103.18 ± 2.87	111.59 ± 0.81
**14b**	27.03 ± 0.98	122.69 ± 2.08	114.06 ± 1.68	125.09 ± 2.78	98.02 ± 0.87	93.20 ± 0.61
**14c**	22.56 ± 0.88	125.49 ± 1.53	109.87 ± 2.45	65.35 ± 4.55	57.23 ± 4.16	104.95 ± 2.38
**14d**	29.01 ± 0.13	141.27 ± 1.17	119.56 ± 2.54	133.61 ± 3.97	90.46 ± 3.81	117.85 ± 2.43
**14e**	46.52 ± 2.68	134.12 ± 0.79	126.72 ± 1.89	117.73 ± 5.23	86.45 ± 4.84	97.53 ± 2.69
**14f**	39.59 ± 1.77	71.43 ± 1.66	74.17 ± 1.07	80.62 ± 0.60	79.77 ± 2.53	90.98 ± 0.38
**14g**	28.23 ± 0.55	75.26 ± 1.09	45.48 ± 0.99	74.81 ± 1.72	87.57 ± 1.30	102.65 ± 4.98
**Gefitinib**	31.11 ± 0.73	55.05 ± 0.76	63.73 ± 0.62	75.25 ± 1.66	84.25 ± 0.32	82.11 ± 0.74

**TABLE 2:** IC_50_ values of new compounds.

Compd no.	IC_50_(μM)	Compound no.	IC_50_(μM)
	48 h		72 h
**13c**	>100	**13c**	63.95 ± 6.77
**13e**	>100	**13e**	64.67 ± 7.35
**13f**	55.25 ± 1.56	**13f**	53.34 ± 6.64
**13h**	51.12 ± 2.54	**13h**	49.02 ± 4.27
**13j**	43.45 ± 5.67	**13j**	35.26 ± 5.81
**13l**	>100	**13L**	79.23 ± 6.11
**13n**	88.58 ± 18.03	**13n**	76.97 ± 6.21
**13q**	29.38 ± 3.49	**13q**	34.91 ± 5.25
**14b**	25.06 ± 4.62	**14b**	7.59 ± 0.31
**14c**	24.05 ± 3.83	**14c**	18.52 ± 0.59
**14d**	38.68 ± 14.89	**14d**	34.80 ± 1.96
**14e**	29.06 ± 1.15	**14e**	27.79 ± 0.96

### 3.2 Cell apoptosis of novel synthetic compounds

**FIGURE 3:**
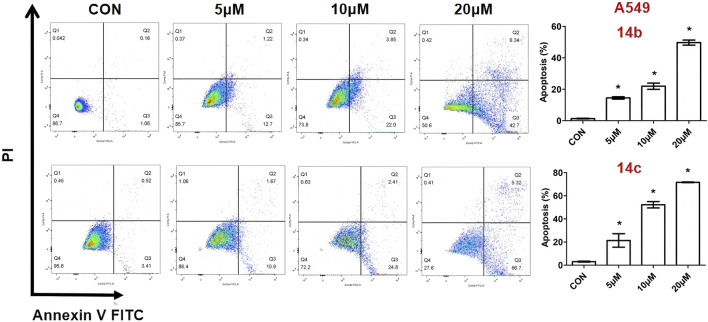
Compounds **14b** and **14c** induced cell apoptosis.

The above results show that compounds **14b** and **14c** exhibited the most effective activity against cancer cell lines, especially A549. We used these two compounds to clarify this function and explore the underlying mechanism. To investigate whether the new compound could induce cell apoptosis, A549 cells were exposed to different concentrations of **14b** and **14c** for 48 h. The cells were then stained with Annexin V-FITC and propidium iodide (PI). The Annexin V-FITC/PI staining was performed to quantify the percentage of apoptotic cells by flow cytometry. The results showed that the percentage of early and late apoptotic cells increased from 1.22% (control) to 13.92% (5 μM), 25.85% (10 μM), and 49.04% (20 μM) after compound **14b** treatment (Figure 5), and from 3.93% (control) to 12.57% (5 μM), 27.21% (10 μM), and 72.02% (20 μM) after compound **14c** treatment (Figure 3). This suggests that **14b** and **14c** compounds could induce cell apoptosis in a dose-dependent manner.

A549 cells were exposed to different concentrations of **14b** and **14c** (5 μM, 10 μM, and 20 μM) for 48 h. Cells were collected and stained with Annexin V-FITC and PI, and apoptotic cells were measured by flow cytometry. Data are presented as mean ± SEM, *P < 0.05.

### 3.3 Compounds 14b and 14c affected cell growth

**FIGURE 4:**
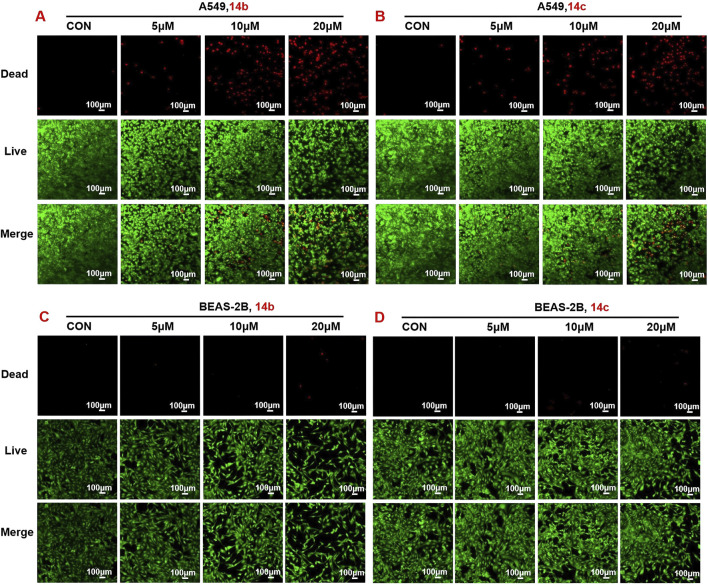
Compound 14b and 14c increased dead cells and decreased live cells. **(A–B)** A549 cells were treated with different concentrations of **14b** and **14c** (5 μM, 10 μM, and 20 μM) for 24 h **(C–D)** BEAS-2B cells were treated with different concentrations of **14b** and **14c** (5 μM, 10 μM, and 20 μM) for 24 h. Live and dead cell numbers were stained with calcein in AM and PI, respectively, and cells were observed by confocal microscopy. Data are presented as mean ± SEM, *P < 0.05.

To investigate the role of **14b** and **14c** in regulating cell proliferation and death, A549 cells were treated with different concentrations of compounds for 24 h, and the live/dead cell numbers were then observed by staining with calcein AM and propidium iodide. Dead cells increased after treatment with 5 μM of **14b** compound and were greatly and largely increased after exposure to 10 μM and 20 μM of compound **14b**. Live cells were consistently decreased after the addition of different concentrations of compound **14b** (Figure 4A). The same results were also observed in compound **14c**-exposed cells (Figure 4B). Furthermore, BEAS-2B cells treated with **14b** and **14c** showed minimal cytotoxicity, suggesting that these compounds had little effect on normal lung cells (Figures 4C, D).

### 3.4 Effects of 2h-1, 4-benzoxazin-3(4H)-one-linked 1,2,3-triazole derivatives on the gene expression of cell growth process

**FIGURE 5:**
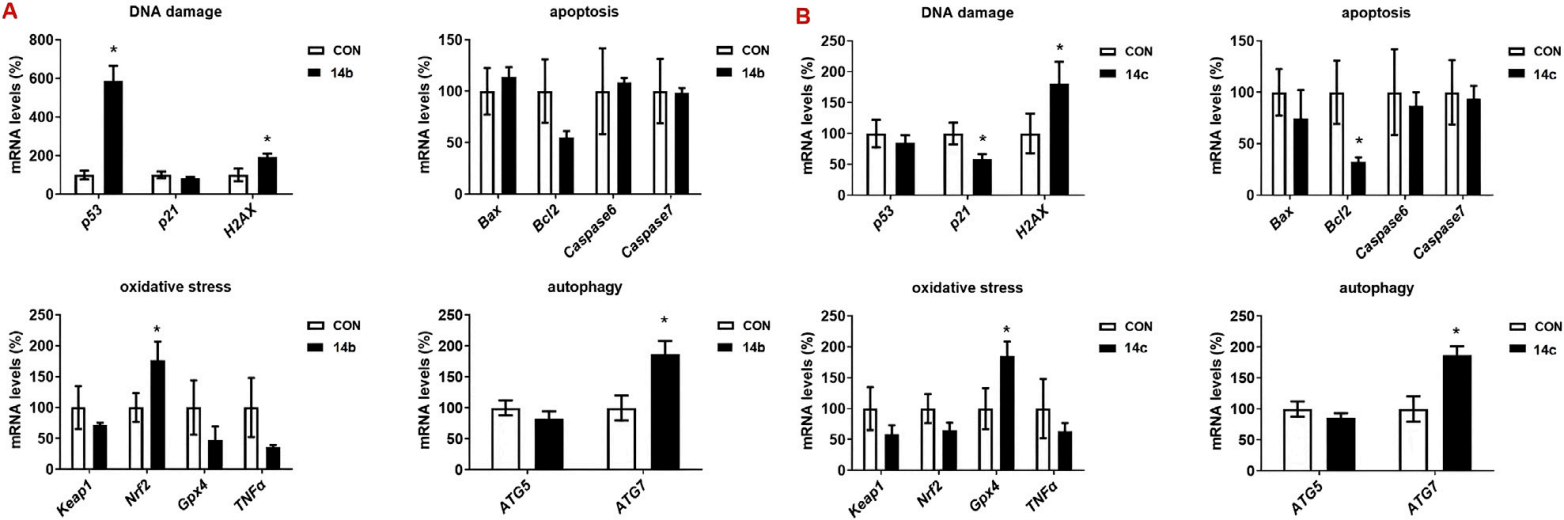
Compounds 14b and 14c increased the gene expression. A549 cells were treated with **14b (A)** and **14c (B)** (10 μM) for 48 h. Gene expressions were detected by RT-PCR. Data are presented as mean ± SEM, *P < 0.05.

To investigate the molecular mechanisms underlying the anti-tumor activity of 2H-1,4-benzoxazin-3(4H)-one-linked 1,2,3-triazole derivatives, we examined the expression of key genes involved in cell growth and death, including those related to DNA damage, oxidative stress, apoptosis, and autophagy (Figure 5). Gene expression was assessed in A549 cells treated with **14b** or **14c** (10 μM) for 48 h. Treatment with **14b** significantly activated DNA damage, oxidative stress, and autophagy pathways, with increased expression of *p53*, *Nrf2*, and *ATG7*. Similarly, **14c** treatment upregulated *H2AX* (DNA damage), *Gpx4* (oxidative stress), and *ATG7* (autophagy), although apoptosis-related genes were either downregulated or unchanged. The regulation of gene expression is a complex process regulated by many signaling pathways, and these signaling pathways interact with each other, all of which lead to a change in gene expression, not all in the same direction. Although the DNA damage gene p21 was decreased, another key gene, H2AX, was increased, which may stimulate DNA damage. These findings in A549 cells align with the results observed following **14b** and **14c** treatments. From these results, we suggest that the mechanism of the anti-tumor effect of the compounds may be caused by increasing DNA damage, oxidative stress, and autophagy.

### 3.5 Compounds 14b and 14c stimulated ROS generation

**FIGURE 6:**
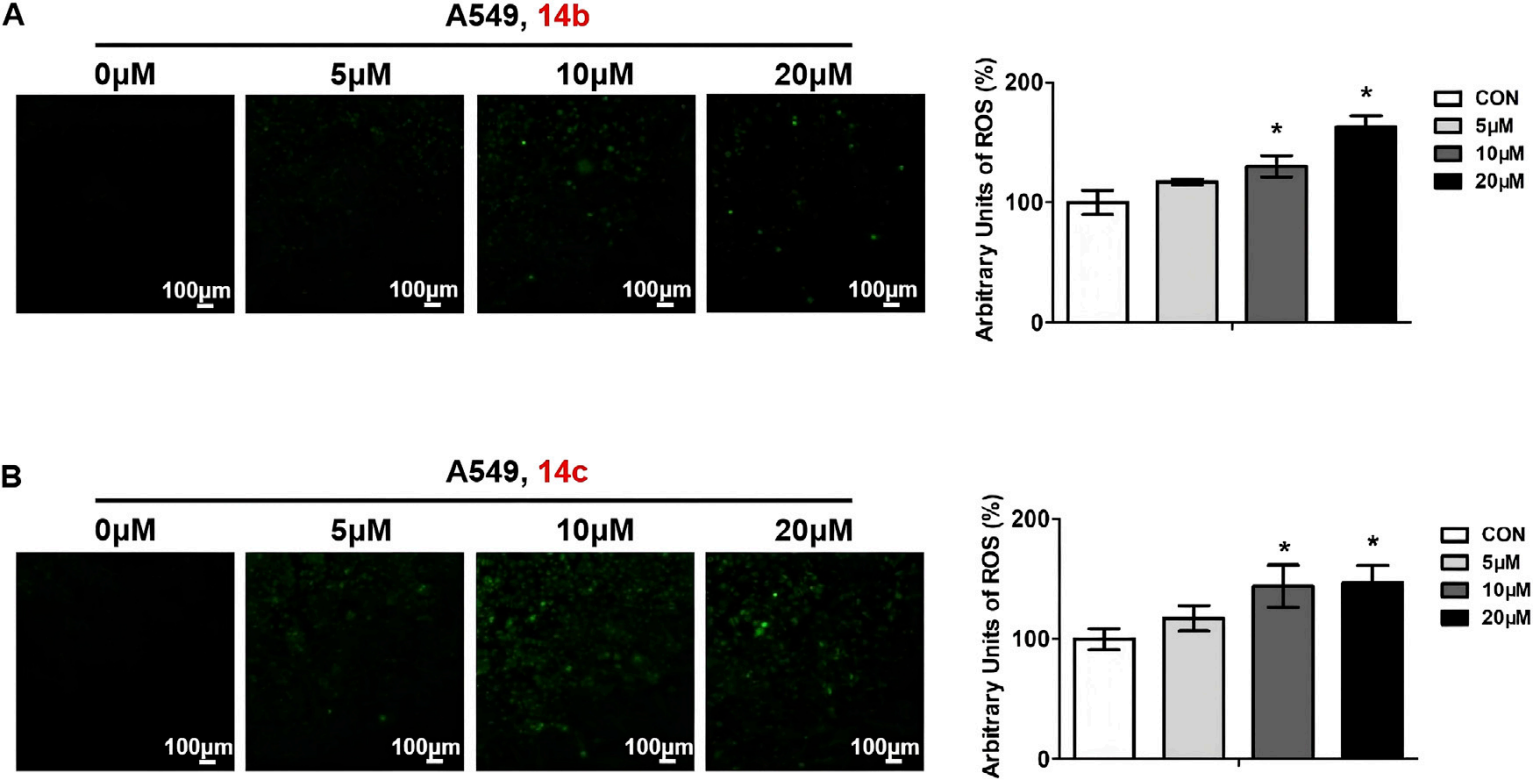
Compounds 14b and 14c increased ROS levels. A549 cells were treated with different concentrations of **14b** **(A)** and **14c** **(B)** (5 μM, 10 μM, or 20 μM) for 24 h. ROS levels were stained using DCFH-DA, observed by confocal microscopy, and analyzed by ImageJ. Data are presented as mean ± SEM, *P < 0.05.

Intracellular reactive oxygen species (ROS) generation will result in mitochondrial membrane damage and further induce cell apoptosis and cell death. To determine whether compounds **14b** and **14c** could induce ROS generation, we treated A549 cells with different doses of these two compounds and measured the intracellular ROS levels using the DCFH-DA peroxide-sensitive fluorescent dye. When DCFH-DA entered the cells, it would hydrolyze into DCFH by esterase and then convert to non-diffusible green fluorescent DCF by oxidation. The results showed that compounds **14b** and **14c** increased ROS generation, and ROS levels increased with the increased concentration to some degree (Figure 6).

### 3.6 Compounds 14b and 14c induced DNA damage and autophagy

**FIGURE 7:**
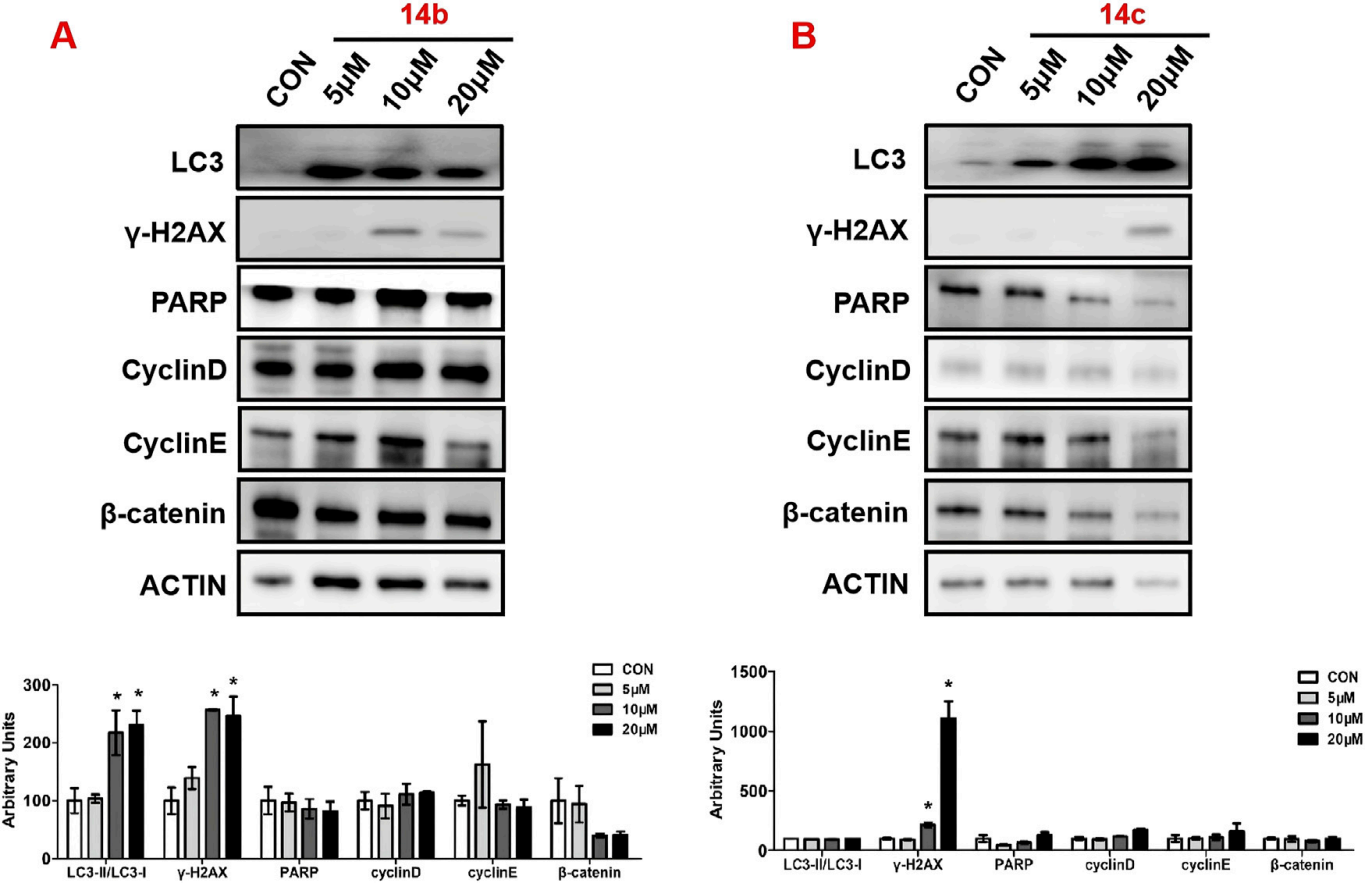
Compounds 14b and 14c affected DNA damage and autophagy. A549 cells were treated with different concentrations of 14b **(A)** and 14c **(B)** (5 μM, 10 μM, and 20 μM) for 48 h. Protein expressions were analyzed by Western blot. Top: Western blot of LC3, γ-H2AX, PARP, cyclin D, cyclin E, and β-catenin. Bottom: quantitative measurements relative to ACTIN. Data are presented as mean ± SEM, *P < 0.05.

Next, we measured the expression of key proteins involved in autophagy, DNA damage, and cell cycle progression in **14b** and **14c** compound-exposed cells. After treatment for 48 h, cell proteins were collected and measured by Western blot. The results showed that compound 14 b significantly promoted autophagy, which was reflected by the increased ratio of LC3II to LC3I (Figure 7). Moreover, the DNA damage progression-related protein H2AX variant histone (γ-H2AX) was also induced, while poly (ADP-ribose) polymerase (PARP) expression was not changed (Figure 7). Proteins in cell cycle progression, including cyclin D, cyclin E, and β-catenin, however, were not affected in the 14 b compound exposure group compared with the control group. The 14c compound displayed different results in **14c** compound-exposed cells, and DNA damage was increased because of the increased expression ofγ-H2AX. However, the key regulators involved in autophagy and cell cycle progression, such as LC3, cyclin D, cyclin E, and β-catenin, were not changed.

### 3.7 2H-1,4-benzoxazin-3(4H)-one-linked 1,2,3-triazole derivatives stimulated DNA damage in tumor cells

**FIGURE 8:**
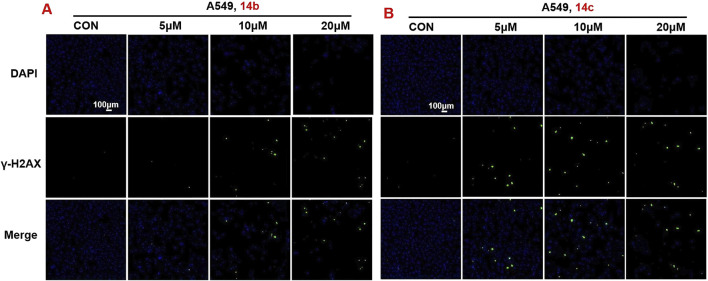
Compounds 14b and 14c affected DNA damage **(A, B)** DNA damage detection shown by γ-H2AX staining in A549 cells treated with compounds 14b and 14c (5 μM, 10 μM, or 20 μM).

In order to further verify the effects of 2H-1,4-benzoxazin-3(4H)-one linked 1,2,3-triazole derivatives on DNA damage, A549 cells were treated with different doses (5, 10, and 20 μM) of compounds **14b** and **14c**. After 24-h treatment, cells were stained with γ-H2AX antibody and DAPI, cell nuclei were stained blue, and DNA-damaged cells were stained green. Using confocal microscopy, cells were observed and photographed. The results showed that compounds **14b** and **14c** significantly led to DNA damage in a concentration-dependent manner, which was consistent with the induced expression of γ-H2AX protein in the above results (Figure 8).

### 3.8 Effects of 2h-1,4-benzoxazin-3(4H)-one-linked 1,2,3-triazole derivatives on autophagy

**FIGURE 9:**
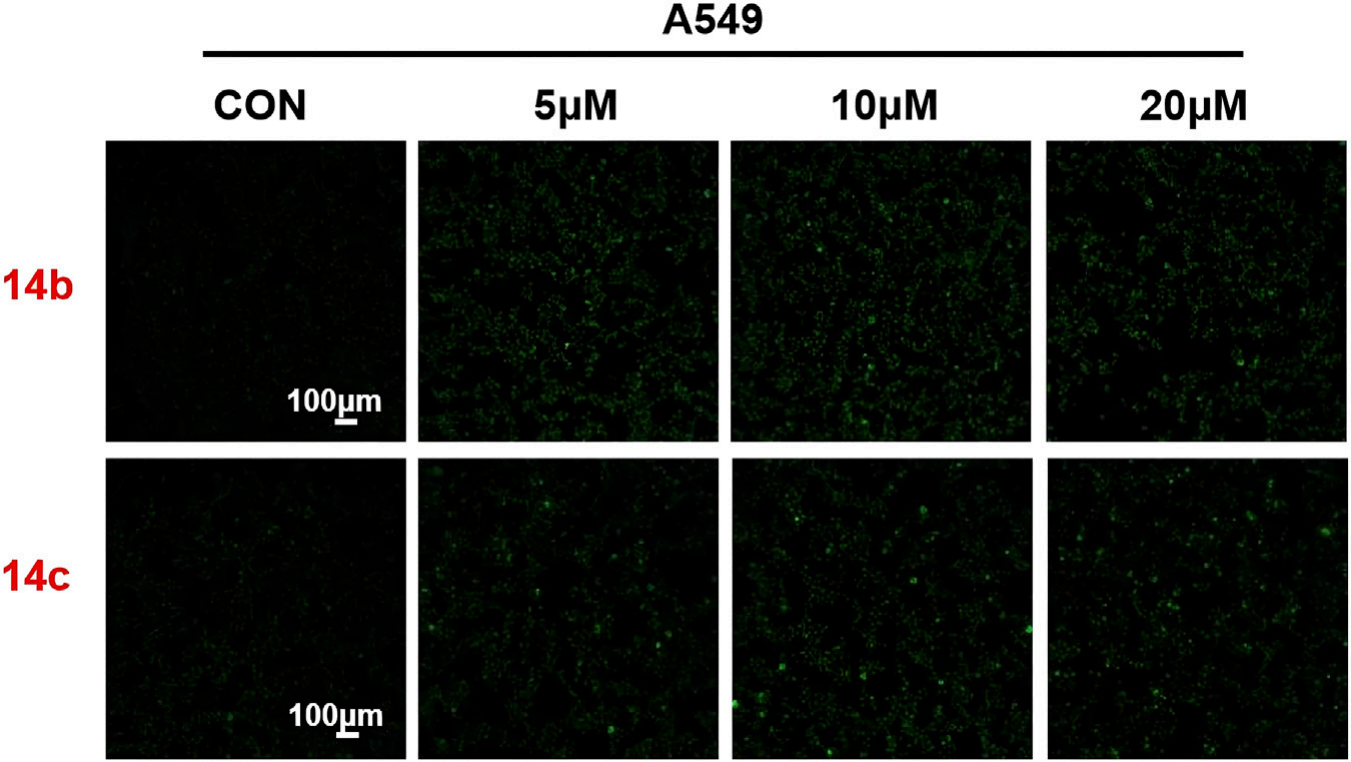
Compounds 14b and 14c affected autophagy. Autophagy staining in A549 cells treated with compounds 14b and 14c (5 μM, 10 μM, or 20 μM).

To further evaluate the role of 2H-1,4-benzoxazin-3(4H)-one-linked 1,2,3-Triazole derivatives in regulating autophagy, A549 cells were treated with different doses (10, 20, and 30 μM) of compounds **14b** and **14c** for 24 h, and the autophagosome was detected by MDC (monodansylcadaverine) probe. MDC could specifically label the autophagosome, which showed as green, and we could directly observe it using a fluorescence microscope. The results indicated that autophagy was largely induced after treatment with compounds **14b** and **14c** (Figure 9), which was consistent with the increased protein expression of LC3. This indicated that 2H-1,4-benzoxazin-3(4H)-one-linked 1,2,3-Triazole derivatives could induce cell autophagy progression.

### 3.9 14b and 14c interaction patterns with DNA in human topoisomerase II beta

**FIGURE 10:**
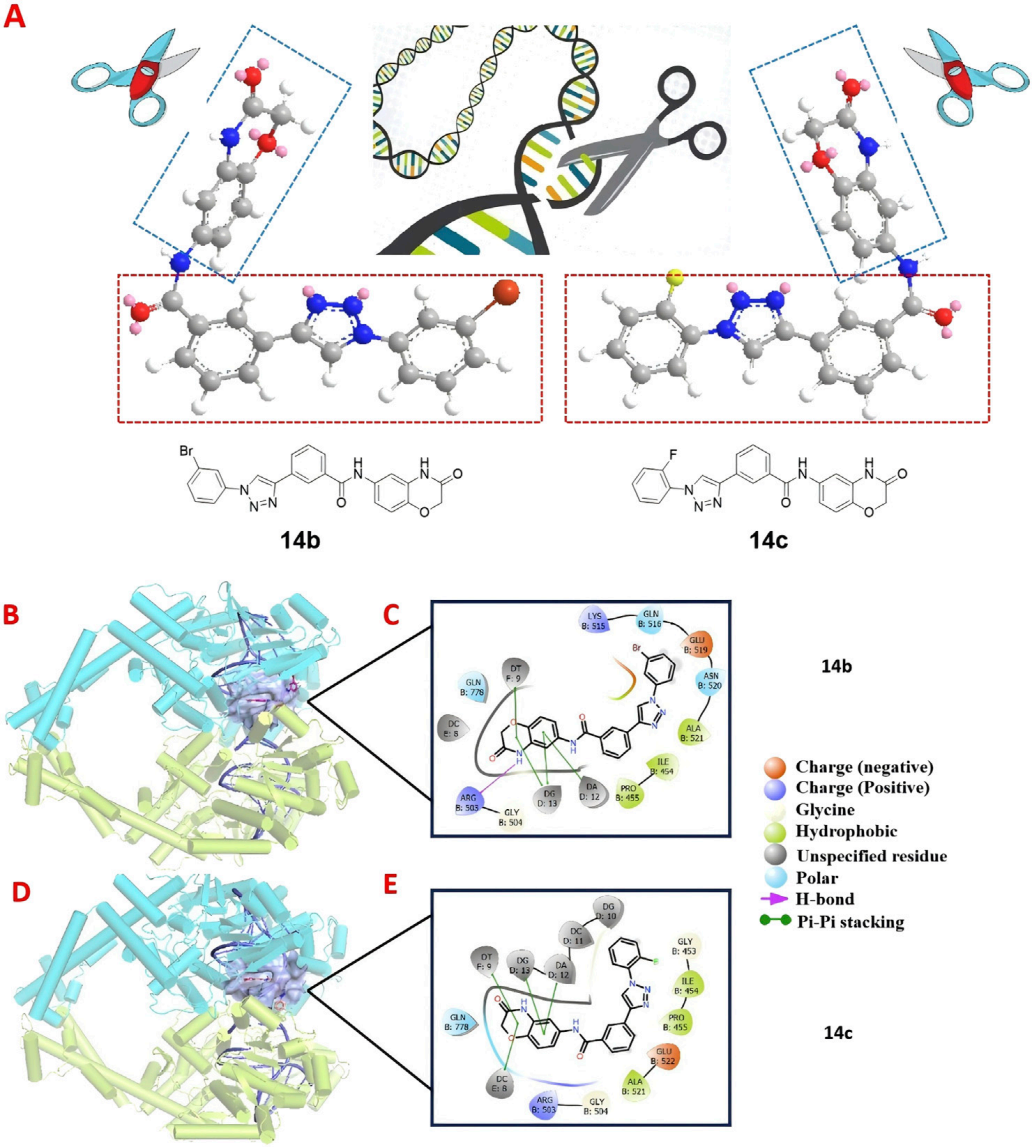
3D molecular structures of compounds **14b** and **14c** along with their docked interactions with DNA in human topoisomerase II, shown in both three- and two-dimensional formats. **(A)** 3D molecular structures of compounds **14b** and **14c**. **(B, C)** Binding modes and 2D schematic diagrams of **14b**, illustrating its critical interactions with DNA. **(D, E)** Binding modes and 2D schematic diagrams of **14c**, highlighting its critical interactions with DNA.

To determine the structural characteristics of compounds **14b** and **14c**, we performed energy minimization on their 3D molecular structures. It can be clearly observed that both **14b** and **14c** consist of two intersecting rigid planar structures (Figure 10A). One planar structure is 2H-1,4-benzoxazin-3(4H)-one, and the other is 1-phenyl-1H-1,2,3-triazole. This configuration allows them to insert into the DNA of tumor cells like a pair of scissors, inducing DNA damage and leading to tumor cell death, thus aligning with our design expectations. Human topoisomerase II plays a critical role in DNA damage and repair, with its polymorphisms contributing to chromosomal instability, tumorigenesis, and the promotion of tumor proliferation and invasiveness in various cancers. Previous studies have reported that some compounds containing rigid planar structures exert their antitumor effects by targeting human topoisomerase II (Kadayat et al., 2015; Mohammed et al., 2022). To further investigate this, we performed molecular docking studies of compounds **14b** and **14c** with human topoisomerase II to examine their interactions with DNA. As shown in Figures 10B–E, the binding site (highlighted in purple) within the DNA of human topoisomerase II beta defines the shape complementarity of the docked ligands. A two-dimensional diagram illustrates the non-bonded interactions between the ligands and the binding site residues. The phthalimide group of **14b** engages in π–π stacking interactions with residues DG D:13, DT F:9, and DT D:12, in addition to forming a hydrogen bond with Arg B:503. Similarly, the phthalimide group of **14c** forms π–π stacking interactions with residues DA D:12, DG D:13, DC E:8, and DT F:9. The molecular docking experiments demonstrated that compounds **14b** and **14c** can induce apoptosis in tumor cells by acting on the DNA of the tumor cells.

### 3.10 Acute toxicity test of compound 14b in mice

We further evaluated the safety of compound **14b** through an acute toxicity study in KM mice. Male and female mice were divided into two groups: a control group and a **14 b-treated** group. The treated group received 800 mg/kg of compound **14b** via oral gavage, while the control group was given an equivalent volume of solvent. Body weight was monitored over a 15-day period. At the end of the study, blood samples were collected from the retro-orbital sinus to assess the biochemical markers GPT and GOT, and the major organs—heart, liver, spleen, lungs, and kidneys—were weighed and subjected to HE staining.

**FIGURE 11:**
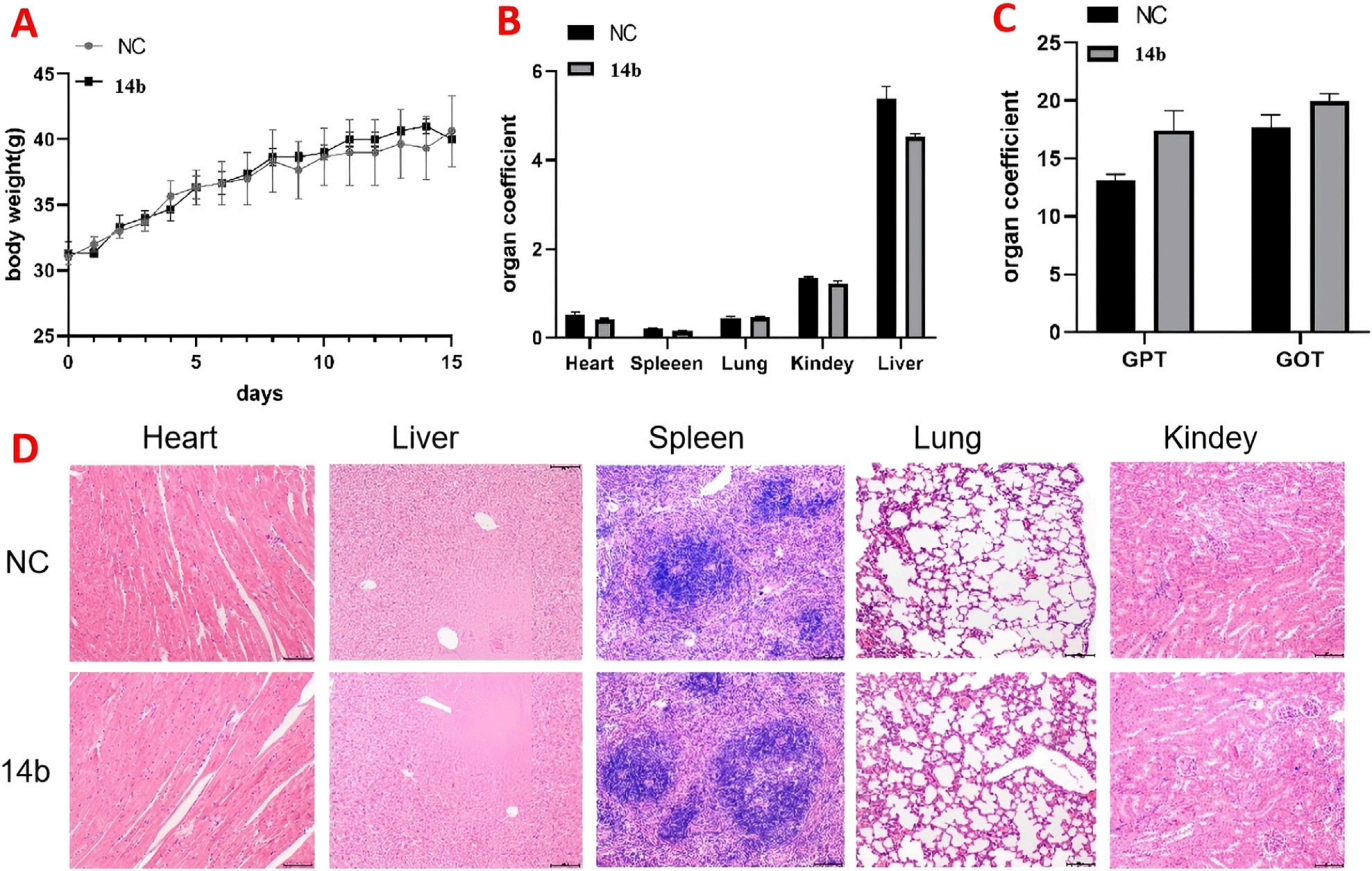
Acute toxicity experiments of compound **14b** were conducted in mice **(A)**. The body weights of the mice were recorded for 14 days after treatment **(B)**. Acute toxicity experiments examined the effects of **14b** on mouse organs **(C)**. Effect of acute toxicity experimental studies on blood biochemical indices GPT and GOT in mice **(D)**. H&E staining was performed on various organs of mice treated with compound **14b**.

As shown in Figure 11A, there were no significant differences in body weight between the **14 b-treated** and control groups. Similarly, Figure 11B shows no significant changes in the organ indices of the major organs between the groups. Biochemical analysis revealed a slight but not statistically significant increase in GPT levels in the 14 b-treated group (Figure 11C). Furthermore, HE staining of the major organs (heart, liver, spleen, lungs, and kidneys) showed no significant histopathological differences between the 14 b-treated and control groups (Figure 11D). These results suggest that compound 14 b has a favorable safety profile, providing experimental support for its potential clinical use.

## 4 Conclusion

A series of 2H-benzo[b][1,4]oxazin-3(4H)-one-linked 1,2,3-triazole compounds were designed and synthesized, and their anticancer abilities were evaluated in several human cancer cell lines. Biological evaluation against various human tumor cell lines revealed that these series’ compounds exhibited significant inhibitory activity against lung cancer cells (A549). Among these, compounds 14b and 14c demonstrated strong anticancer effects, with IC_50_ values of 7.59 μM and 18.52 μM, respectively. Apoptosis analysis in A549 cells further confirmed that 14b and 14c significantly induced apoptosis. The anti-proliferative activity was corroborated by calcein-AM and PI staining, which revealed a marked increase in dead cells and a corresponding decrease in live cells following treatment. Additionally, Western blot analysis showed that 14b and 14c triggered DNA damage and autophagy, with further mechanistic studies suggesting that these compounds may act by intercalating into tumor cell DNA, causing DNA damage. Notably, compound 14b exhibited low toxicity in mice, highlighting its potential as a targeted DNA-damaging anti-tumor agent.

## 5 Experimental section

### 5.1 Materials and chemistry

2H-benzo[b][1,4]oxazin-3(4H)-one-linked 1,2,3-triazole derivatives were synthesized in-house. Dimethyl sulfoxide (DMSO) was obtained from Sigma-Aldrich (St. Louis, Missouri, United States). Dulbecco’s modified Eagle medium (DMEM), RPMI 1640 medium, fetal bovine serum (FBS), and penicillin/streptomycin were purchased from Gibco (Grand Island, NY, United States). The CCK-8 assay kit, Calcein/PI Live/Dead Viability Assay Kit, and Giemsa dye and Reactive Oxygen Species (ROS) Assay Kits were purchased from Beyotime Biotechnology, Shanghai, China. TRIzol reagent was purchased from Ambion, United States. RT-PCR was performed using PowerUp™ SYBR™ Green Master Mix (ABI, United States). Annexin V-FITC/propidium iodide (PI) staining kit and Matrigel Matrix were provided by BD Biosciences (Franklin Lakes, New Jersey, United States). LC3, γ-H2AX, PARP, cyclin D, cyclin E, and β-Catenin were purchased from Cell Signaling Technology, United States, and β-actin was purchased from Sigma, United States.

#### 5.1.1 General synthetic procedure for compounds 13a**–**13r and 14a**–**14e

Compound 11 (0.02 mol), 3-ethynylbenzoic acid (0.03 mol), HATU (0.03 mol), DIPEA (0.06 mol), and 250 mL of DMF were added to a 500 mL reaction flask at room temperature and stirred under nitrogen for 24 h, monitored by TLC. After the reaction was completed, the DMF was removed by vacuum evaporation. The reaction mixture was extracted with dichloromethane (150 mL × 3), and the combined organic extracts were washed with saturated sodium chloride (150 mL × 2) to pH 7. The resulting brownish–yellow viscous liquid was concentrated under vacuum. Methanol was added dropwise under ultrasonic agitation, causing the solid to precipitate. After standing, the solid was filtered, dried, and compound 12 was obtained (Hou et al., 2024).

In a reaction flask, compound 12 (1 eq), substituted azide (1.2 eq), tert-butanol water tetrahydrofuran anhydrous copper sulfate (0.2 eq), and sodium ascorbate (0.4 eq) were added sequentially. The mixture was stirred and refluxed at 70°C for 6 h, monitored by TLC. After the reaction, the mixture was extracted with dichloromethane (30 mL × 3), and the organic solution was washed with saturated sodium chloride (30 mL × 2). The combined organic layer was further washed with brine (30 mL × 2), dried over sodium sulfate, and concentrated under vacuum to yield the crude product. The desired compound was obtained by recrystallization from ethyl acetate and was pure enough for further characterization and anti-tumor studies.

The spectroscopic characterization of compounds **13a–13t** and **14a–14g** is provided as follows.

Compound 13a: m. p. 238.7–241.2^o^C. IR (KBr, cm^-1^): 3,361, 1,690, 1,523, 1,489, 625. HR-MS(ESI): Calcd. C24H18BrN5O3 [M + H]^+^ *m/z*: 504.0671, found: 504.0657. ^1^H NMR (400MHz, DMSO-d_6_): 10.78 (s, 1H), 10.29 (s, 1H), 8.72 (s, 1H), 8.38 (s, 1H), 8.04 (d, J = 8.0Hz, 1H), 7.88 (d, J = 8.0Hz, 1H), 7.62–7.53 (m, 4H), 7.34 (d, J = 8.0Hz, 2H), 7.26 (d, J = 12.0Hz, 1H), 6.93 (d, J = 12.0Hz, 1H), 5.67 (s, 2H), 4.55 (s, 2H). ^13^C NMR (100MHz, DMSO-d_6_): 165.55, 146.65, 139.94, 136.18, 135.79, 134.30, 132.24, 131.25, 130.71, 129.49, 128.50, 127.60, 127.56, 124.88, 122.57, 121.98, 116.40, 115.66, 109.00, 67.32, 52.86.

Compound 13b: m. p. 217.9–221.2^o^C. IR (KBr, cm^-1^): 3,261, 1,697, 1,553, 1,438, 864. HR-MS(ESI): Calcd. C26H23N5O3 [M + H]^+^ *m/z*: 454.1879, found: 454.1964. ^1^H NMR (400MHz, DMSO-d_6_): 10.78 (s, 1H), 10.29 (s, 1H), 8.69 (s, 1H), 8.39 (s, 1H), 8.05 (d, J = 8.0Hz, 1H), 7.88 (d, J = 8.0Hz, 1H), 7.59–7.54 (m, 2H), 7.26 (d, J = 12.0Hz, 1H), 6.98–6.92 (m, 4H), 5.58 (s, 2H), 4.55 (s, 2H), 2.26 (s, 6H). ^13^C NMR (100MHz, DMSO-d_6_): 165.55, 146.56, 139.94, 138.43, 136.16, 134.30, 131.33, 130.08, 129.47, 128.48, 127.60, 127.50, 126.18, 124.84, 122.42, 116.40, 115.66, 109.01, 67.32, 53.61, 21.30.

Compound 13c: m. p. 208.2–211.3^o^C. IR (KBr, cm^-1^):3,434. 1,693, 1,588, 1,452, 1,281. HR-MS(ESI): Calcd. C24H16F3N5O3 [M + H]^+^ *m/z*: 480.1283, found: 480.1282. ^1^H NMR (400MHz, DMSO-d_6_): 10.78 (s, 1H), 10.30 (s, 1H), 8.74 (s, 1H), 8.38 (s, 1H), 8.04 (d, J = 8.0Hz, 1H), 7.89 (d, J = 12.0Hz, 1H), 7.62–7.58 (m, 1H), 7.54 (s, 1H), 7.43–7.39 (m, 2H), 7.27–7.24 (m, 1H), 6.94 (d, J = 8.0Hz, 1H), 5.58 (s, 2H), 4.55 (s, 2H). ^13^C NMR (100MHz, DMSO-d_6_): 165.55, 146.67, 139.94, 136.20, 134.29, 131.19, 129.48, 128.53, 127.60, 124.94, 122.68, 113.85, 113.79, 113.69, 113.63, 108.99, 67.31, 60.21, 52.19, 21.22, 14.55.

Compound 13d: m. p. 216.2–220.9^o^C. IR (KBr, cm^-1^):3,450, 2,228, 1,683, 1,585, 1,493, 1,219. HR-MS(ESI): Calcd. C25H17FN6O3 [M + H]^+^ *m/z*: 469.1424, found: 469.1412. ^1^H NMR (400MHz, DMSO-d_6_): 10.78 (s, 1H), 10.31 (s, 1H), 8.77 (s, 1H), 8.39 (s, 1H), 8.07–8.05 (m, 2H), 7.90 (d, J = 12.0Hz, 1H), 7.63–7.59 (m, 1H), 7.5–7.41 (m, 3H), 7.27–7.24 (m, 1H), 6.94 (d, J = 8.0Hz, 1H), 5.91 (s, 2H), 4.55 (s, 2H). ^13^C NMR (100MHz, DMSO-d_6_): 165.56, 146.59, 142.40, 139.94, 137.01, 136.91, 136.22, 134.30, 131.10, 129.50, 128.58, 127.60, 124.97, 123.05, 118.02, 117.78, 117.49, 117.27, 116.74, 116.40, 115.66, 109.00, 108.48, 67.32, 51.50.

Compound 13e: m. p. 245.8–246.2^o^C. IR (KBr, cm^-1^):3,403, 1,695, 1,581, 1,450, 1,248, 697. HR-MS(ESI): Calcd. C24H17ClFN5O3 [M + H]^+^ *m/z*: 478.1082, found: 478.1063. ^1^H NMR (400MHz, DMSO-d_6_): 10.78 (s, 1H), 10.28 (s, 1H), 8.69 (s, 1H), 8.38 (s, 1H), 8.06 (d, J = 8.0Hz, 1H), 7.87 (d, J = 8.0Hz, 1H), 7.60–7.52 (m, 3H), 7.46–7.35 (m, 2H), 7.27–7.24 (m, 1H), 6.94 (d, J = 12.0Hz, 1H), 5.79 (s, 2H), 4.55 (s, 2H). ^13^C NMR (100MHz, DMSO-d_6_): 165.55, 146.24, 139.93, 136.20, 135.52, 134.30, 132.43, 132.33, 131.16, 129.43, 128.58, 127.59, 126.38, 124.93, 122.61, 116.40, 115.66, 115.39, 109.00, 67.32, 45.22.

Compound 13f: m. p. 227.7–229.8^o^C. IR (KBr, cm^-1^): 3,360, 1722, 1,524, 1,440, 1,225, 694. HR-MS(ESI): Calcd. C24H17ClFN5O3 [M + H]^+^ *m/z*: 478.1082, found: 478.1062. ^1^H NMR (400MHz, DMSO-d_6_): 10.78 (s, 1H), 10.30 (s, 1H), 8.75 (s, 1H), 8.38 (s, 1H), 8.04 (d, J = 12.0Hz, 1H), 7.89 (d, J = 8.0Hz, 1H), 7.69–7.67 (m, 1H), 7.60 (t, J_1_ = 8.0Hz, J_2_ = 8.0Hz, 1H), 7.54 (s, 1H), 7.46–7.42 (m, 2H), 7.25 (dd, J_1_ = 4.0Hz, J_2_ = 4.0Hz, 1H), 6.94 (d, J = 8.0Hz, 1H), 5.69 (s, 2H), 4.55 (s, 2H). ^13^C NMR (100MHz, DMSO-d_6_): 165.55, 146.67, 139.94, 136.19, 134.30, 130.98, 129.61, 129.53, 129.48, 128.52, 127.57, 124.90, 122.57, 120.29, 120.11, 117.94, 117.73, 116.40, 115.65, 109.00, 67.32, 52.24.

Compound 13g: m. p. 223.2–226.9^o^C. IR (KBr, cm^-1^): 3,361, 1,694, 1,550, 1,476, 748. HR-MS(ESI): Calcd. C24H18ClN5O3 [M + H]^+^ *m/z*: 460.1176, found: 460.1163. ^1^H NMR (400MHz, DMSO-d_6_): 10.78 (s, 1H), 10.30 (s, 1H), 8.76 (s, 1H), 8.39 (s, 1H), 8.05 (d, J = 12.0Hz, 1H), 7.89 (d, J = 8.0Hz, 1H), 7.60 (t, J_1_ = 8.0Hz, J_2_ = 8.0Hz, 1H), 7.54 (s, 1H), 7.48–7.43 (m, 3H), 7.35–7.32 (m, 1H), 7.26 (dd, J_1_ = 4.0Hz, J_2_ = 4.0Hz, 1H), 6.94 (d, J = 8.0Hz, 1H), 5.71 (s, 2H), 4.55 (s, 2H). ^13^C NMR (100MHz, DMSO-d_6_): 165.55, 146.67, 139.94, 138.75, 136.19, 134.30, 133.84, 131.25, 129.49, 128.70, 128.52, 128.38, 127.60, 127.19, 124.90, 122.66, 116.40, 115.65, 109.00, 67.32, 52.83.

Compound 13h: m. p. 231.9–232.8^o^C. IR (KBr, cm^-1^): 3,407, 1,696, 1,519, 1,489, 1,215, 751. HR-MS(ESI): Calcd. C24H17ClFN5O3 [M + H]^+^ *m/z*: 478.1082, found: 478.1063. ^1^H NMR (400MHz, DMSO-d_6_): 10.78 (s, 1H), 10.30 (s, 1H), 8.74 (s, 1H), 8.39 (s, 1H), 8.07 (s, 1H), 7.88 (s, 1H), 7.61–7.25 (m, 6H), 6.94 (d, J = 8.0Hz, 1H), 5.74 (s, 2H), 4.55 (s, 2H). ^13^C NMR (100MHz, DMSO-d_6_): 165.56, 160.72, 158.26, 146.55, 139.94, 138.59, 136.20, 134.30, 131.15, 131.06, 129.49, 128.55, 127.60, 125.29, 125.12, 124.92, 122.69, 118.34, 118.10, 116.40, 115.67, 109.02, 67.32, 47.32.

Compound 13i: m. p. 217.2–220.6^o^C. IR (KBr, cm^-1^): 3,360, 1722, 1,523, 1,477, 1,225. HR-MS(ESI): Calcd. C24H18FN5O3 [M + H]^+^ *m/z*: 444.1472, found: 444.1479. ^1^H NMR (400MHz, DMSO-d_6_): 10.80 (s, 1H), 10.31 (s, 1H), 8.73 (s, 1H), 8.38 (s, 1H), 8.06–8.04 (m, 1H), 7.88 (d, J = 8.0Hz, 1H), 7.62–7.44 (m, 4H), 7.27–7.24 (m, 2H), 6.94 (d, J = 8.0Hz, 1H), 5.68 (s, 2H), 4.56 (s, 2H). ^13^C NMR (100MHz, DMSO-d_6_): 165.58, 163.64, 161.26, 146.64, 139.83, 136.15, 135.87, 134.29, 132.62, 131.26, 129.50, 128.49, 127.58, 124.85, 122.44, 116.42, 116.25, 116.04, 115.65, 108.98, 67.30, 52.80.

Compound 13j: m. p. 232.0–225.2^o^C. IR (KBr, cm^-1^): 3,335, 1,682, 1,615, 1,493, 1,216, 757. HR-MS(ESI): Calcd. C24H18FN5O3 [M + H]^+^ *m/z*: 444.1472, found: 444.1465. ^1^H NMR (400MHz, DMSO-d_6_): 10.78 (s, 1H), 10.29 (s, 1H), 8.71 (s, 1H), 8.39 (s, 1H), 8.04 (d, J = 8.0Hz, 1H), 7.88 (d, J = 8.0Hz, 1H), 7.61–7.26 (m, 7H), 6.94 (d, J = 8.0Hz, 1H), 5.74 (s, 2H), 4.55 (s, 1H). ^13^C NMR (100MHz, DMSO-d_6_): 165.56, 146.52, 139.94, 136.18, 134.30, 131.27, 129.48, 128.54, 127.60, 125.41, 124.89, 123.24, 123.09, 122.59, 116.40, 116.26, 116.05, 115.67, 109.02, 67.32, 47.69.

Compound 13k: m. p. 210.9–214.3^o^C. IR (KBr, cm^-1^): 3,209, 1,688, 1,558, 1,491, 525. HR-MS(ESI): Calcd. C24H17Br2N5O3 [M + H]^+^ *m/z*: 581.9776, found: 581.9769. ^1^H NMR (400MHz, DMSO-d_6_): 10.78 (s, 1H), 10.30 (s, 1H), 8.78 (s, 1H), 8.39 (s, 1H), 8.06 (d, J = 8.0Hz, 1H), 7.91–7.82 (m, 2H), 7.64–7.59 (m, 3H), 7.55 (d, J = 4.0Hz, 1H), 7.27–7.25 (m, 1H), 6.94 (d, J = 8.0Hz, 1H), 5.71 (s, 2H), 4.55 (s, 1H). ^13^C NMR (100MHz, DMSO-d_6_): 165.55, 146.70, 140.74, 139.94, 136.20, 134.30, 133.76, 130.66, 129.51, 128.55, 127.60, 124.93, 123.23, 122.80, 116.41, 115.66, 109.00, 67.32, 52.41, 52.08, 49.08.

Compound 13L: m. p. 128.0–132.7^o^C. IR (KBr, cm^-1^): 3,439, 1,693, 1,542, 1,488, 1,219, 515. HR-MS(ESI): Calcd. C24H17BrFN5O3 [M + H]^+^ *m/z*: 522.0577, found: 522.0571. ^1^H NMR (400MHz, DMSO-d_6_): 10.77 (s, 1H), 10.29 (s, 1H), 8.70 (s, 1H), 8.38 (s, 1H), 8.05 (d, J = 8.0Hz, 1H), 7.88 (d, J = 8.0Hz, 1H), 7.66–7.37 (m, 5H), 7.25 (d, J = 8.0Hz, 1H), 6.93 (d, J = 8.0Hz, 1H), 5.72 (s, 2H), 4.55 (s, 1H). ^13^C NMR (100MHz, DMSO-d_6_): 165.55, 146.54, 139.94, 136.19, 134.29, 132.92, 132.88, 131.18, 129.48, 128.61, 128.58, 128.53, 127.59, 124.89, 122.82, 122.62, 119.76, 119.52, 116.40, 115.60, 109.01, 67.32, 47.29.

Compound 13m: m. p. 200.2–203.9^o^C. IR (KBr, cm^-1^): 3,275, 1,697, 1,554, 1,496, 1,217. HR-MS(ESI): Calcd. C25H18F3N5O3 [M + H]^+^ *m/z*: 494.1440, found: 494.1429. ^1^H NMR (400MHz, DMSO-d_6_): 10.78 (s, 1H), 10.30 (s, 1H), 8.79 (s, 1H), 8.39 (s, 1H), 8.06–7.55 (m, 8H), 7.26 (d, J = 8.0Hz, 1H), 6.94 (d, J = 8.0Hz, 1H), 5.82 (s, 2H), 4.55 (s, 2H). ^13^C NMR (100MHz, DMSO-d_6_): 165.54, 139.95, 137.79, 136.19, 134.30, 132.65, 131.21, 130.52, 129.50, 128.53, 127.60, 125.53, 125.48, 125.18, 124.90, 122.72, 116.40, 115.66, 109.00, 67.32, 52.87.

Compound 13n: m. p. 257.6–261.6^o^C. IR (KBr, cm^-1^): 3,431, 1,697, 1,519, 1,490, 692. HR-MS(ESI): Calcd. C24H17Cl2N5O3 [M + H]^+^ *m/z*: 494.0787, found: 494.0780. ^1^H NMR (400MHz, DMSO-d_6_): 10.78 (s, 1H), 10.28 (s, 1H), 8.65 (s, 1H), 8.38 (s, 1H), 8.06 (d, J = 8.0Hz, 1H), 7.86 (d, J = 12.0Hz, 1H), 7.62–7.48 (m, 5H), 7.25 (d, J = 8.0Hz, 1H), 6.93 (d, J = 8.0Hz, 1H), 5.88 (s, 2H), 4.55 (s, 2H). ^13^C NMR (100MHz, DMSO-d_6_): 165.61, 165.55, 146.10, 139.92, 136.53, 136.22, 134.30, 132.19, 131.17, 130.64, 129.51, 129.42, 128.58, 127.59, 127.54, 124.94, 122.53, 116.39, 115.65, 108.99, 67.32, 49.39.

Compound 13o: m. p. 211.0–212.1^o^C. IR (KBr, cm^-1^): 3,278, 2,226, 1,692, 1,519, 1,489. HR-MS(ESI): Calcd. C25H18N6O3 [M + H]^+^ *m/z*: 451.1519, found: 451.1507. ^1^H NMR (400MHz, DMSO-d_6_): 10.79 (s, 1H), 10.31 (s, 1H), 8.77 (s, 1H), 8.40 (s, 1H), 8.07 (d, J = 8.0Hz, 1H), 7.92 (dd, J_1_ = 8.0Hz, J_2_ = 8.0Hz, 2H), 7.77 (t, J_1_ = 8.0Hz, J_2_ = 8.0Hz, 1H), 7.63–7.47 (m, 4H), 7.28–7.25 (m, 1H), 6.94 (d, J = 8.0Hz, 1H), 5.91 (s, 2H), 4.56 (s, 2H). ^13^C NMR (100MHz, DMSO-d_6_): 165.55, 146.57, 139.94, 139.05, 136.21, 134.37, 134.30, 133.92, 131.15, 130.06, 129.78, 129.51, 128.56, 127.60, 124.94, 122.98, 117.44, 116.40, 115.66, 111.79, 109.01, 67.32, 51.82.

Compound 13p: m. p. 227.2–231.0^o^C. IR (KBr, cm^-1^): 3,361, 1723, 1,549, 1,476, 1,175. HR-MS(ESI): Calcd. C25H18F3N5O3 [M + H]^+^ *m/z*: 494.1440, found: 494.1435. ^1^H NMR (400MHz, DMSO-d_6_): 10.78 (s, 1H), 10.30 (s, 1H), 8.78 (s, 1H), 8.39 (s, 1H), 8.06 (d, J = 12.0Hz, 1H), 7.91–7.88 (m, 1H), 7.78 (d, J = 8.0Hz, 2H), 7.60–7.54 (m, 4H), 7.25 (dd, J_1_ = 4.0Hz, J_2_ = 4.0Hz, 1H), 6.94 (d, J = 8.0Hz, 1H), 5.82 (s, 2H), 4.55 (s, 2H). ^13^C NMR (100MHz, DMSO-d_6_): 165.55, 146.72, 141.06, 139.94, 136.19, 134.30, 131.22, 129.50, 129.39, 129.14, 129.08, 128.52, 127.60, 126.25, 126.21, 124.91, 122.80, 116.40, 115.65, 109.00, 67.32, 52.93.

Compound 13q: m. p. 205.4–220.5^o^C. IR (KBr, cm^-1^): 3,496, 1,697, 1,555, 1,495, 521. HR-MS(ESI): Calcd. C24H18BrN5O3 [M + H]^+^ *m/z*: 504.0671, found: 504.0666. ^1^H NMR (400MHz, DMSO-d_6_): 10.80 (s, 1H), 10.31 (s, 1H), 8.70 (s, 1H), 8.40 (s, 1H), 8.07 (d, J = 8.0Hz, 1H), 7.89 (d, J = 8.0Hz, 1H), 7.72 (d, J = 8.0Hz, 2H), 7.62–7.24 (m, 6H), 6.94 (d, J = 8.0Hz, 1H), 5.77 (s, 2H), 4.55 (s, 2H). ^13^C NMR (100MHz, DMSO-d_6_): 165.55, 146.41, 139.92, 136.18, 135.17, 134.30, 133.45, 131.22, 131.12, 131.00, 129.50, 128.84, 128.55, 127.59, 124.88, 123.45, 122.87, 122.55, 116.41, 115.63, 108.97, 67.30, 53.71.

Compound 13r: m. p. 211.8–216.8^o^C. IR (KBr, cm^-1^): 3,279, 1701, 1,589, 1,492, 1,218. HR-MS(ESI): Calcd. C24H18FN5O3 [M + H]^+^ *m/z*: 444.1472, found: 444.1473. ^1^H NMR (400MHz, DMSO-d_6_): 10.77 (s, 1H), 10.29 (s, 1H), 8.75 (s, 1H), 8.38 (s, 1H), 8.05 (d, J = 12.0Hz, 1H), 7.88 (d, J = 8.0Hz, 1H), 7.59 (t, J_1_ = 8.0Hz, J_2_ = 8.0Hz, 1H), 7.54 (d, J = 4.0Hz, 1H), 7.48–7.43 (m, 1H), 7.26–7.17 (m, 4H), 6.93 (d, J = 8.0Hz, 1H), 5.71 (s, 2H), 4.54 (s, 2H). ^13^C NMR (100MHz, DMSO-d_6_): 165.55, 146.66, 139.94, 139.07, 136.19, 134.30, 131.45, 131.37, 131.25, 129.49, 128.52, 127.60, 127.56, 124.89, 124.56, 124.54, 122.65, 116.40, 115.65, 115.47, 115.26, 109.00, 67.32, 52.93.

Compound 13s: m. p. 258.3–263.7^o^C. IR (KBr, cm^-1^): 3,323, 1700, 1,524, 1,452, 529. HR-MS(ESI): Calcd. C24H18IN5O3 [M + H]^+^ *m/z*: 552.0533, found: 552.0547. ^1^H NMR (400MHz, DMSO-d_6_): 10.77 (s, 1H), 10.28 (s, 1H), 8.71 (s, 1H), 8.37 (s, 1H), 8.04 (d, J = 12.0Hz, 1H), 7.88 (d, J = 8.0Hz, 1H), 7.11 (d, J = 8.0Hz, 2H), 7.61–7.53 (m, 2H), 7.26–7.17 (m, 3H), 6.93 (d, J = 8.0Hz, 1H), 5.65 (s, 2H), 4.54 (s, 2H). ^13^C NMR (100MHz, DMSO-d_6_): 165.57, 165.54, 146.63, 139.92, 138.09, 136.16, 134.30, 131.24, 130.74, 129.50, 128.49, 127.59, 127.55, 124.85, 122.57, 116.42, 115.62, 108.96, 95.03, 67.30, 53.00.

Compound 13t: m. p. 246.0–251.4^o^C. IR (KBr, cm^-1^): 3,243, 1,695, 1,518, 1,492, 1,214, 723. HR-MS(ESI): Calcd. C24H17ClFN5O3 [M + H]^+^ *m/z*: 478.1082, found: 478.1095. ^1^H NMR (400MHz, DMSO-d_6_): 10.80 (s, 1H), 10.31 (s, 1H), 8.69 (s, 1H), 8.38 (s, 1H), 8.07–8.04 (m, 1H), 7.88 (d, J = 8.0Hz, 1H), 7.61–7.47 (m, 4H), 7.34–7.24 (m, 2H), 6.94 (d, J = 8.0Hz, 1H), 5.77 (s, 2H), 4.55 (s, 2H). ^13^C NMR (100MHz, DMSO-d_6_): 165.58, 163.63, 146.39, 139.92, 136.17, 134.28, 133.18, 133.08, 131.19, 129.95, 129.49, 128.54, 127.58, 124.87, 122.71, 116.42, 115.64, 116.58, 115.37, 108.97, 67.30, 50.79.

Compound 14a: m. p. 260.7–263.4^o^C. IR (KBr, cm^-1^): 3,321, 1,692, 1,526, 1,497, 1,121. HR-MS(ESI): Calcd. C24H16F3N5O3 [M + H]^+^ *m/z*: 480.1283, found: 480.1269. ^1^H NMR (400MHz, DMSO-d_6_): 10.80 (s, 1H), 10.36 (s, 1H), 9.59 (s, 1H), 8.51 (s, 1H), 8.23 (d, J = 8.0Hz, 2H), 8.16 (d, J = 8.0Hz, 1H), 8.05 (d, J = 8.0Hz, 2H), 7.96 (d, J = 8.0Hz, 1H), 7.68 (t, J_1_ = 8.0Hz, J_2_ = 8.0Hz, 1H), 7.56 (s, 1H), 7.28 (d, J = 8.0Hz, 1H), 6.95 (d, J = 8.0Hz, 1H), 4.56 (s, 2H). ^13^C NMR (100MHz, DMSO-d_6_): 165.56, 165.53, 147.57, 139.97, 139.86, 136.40, 134.29, 130.66, 129.63, 128.69, 128.01, 127.94, 127.82, 127.78, 127.62, 120.92, 120.85, 116.42, 115.67, 109.01, 67.32.

Compound 14b: m. p. 231.7–235.2^o^C. IR (KBr, cm^-1^): 3,122, 1,693, 1,588, 1,489, 519. HR-MS(ESI): Calcd. C23H16BrN5O3 [M + H]^+^ *m/z*: 490.0515, found: 490.0556. ^1^H NMR (400MHz, DMSO-d_6_): 10.79 (s, 1H), 10.35 (s, 1H), 9.51 (s, 1H), 8.49 (s, 1H), 8.25 (s, 2H), 8.13 (d, J = 12.0Hz, 1H), 8.05 (d, J = 8.0Hz, 1H), 7.95 (t, J_1_ = 4.0Hz, J_2_ = 4.0Hz, 1H), 7.54 (d, J = 8.0Hz, 1H), 7.67–7.61 (m, 2H), 7.56 (s, 1H), 7.29–7.26 (m, 1H), 6.95 (d, J = 8.0Hz, 1H), 4.56 (s, 2H). ^13^C NMR (100MHz, DMSO-d_6_): 165.56, 150.86, 147.36, 139.97, 138.19, 136.38, 134.30, 132.42, 131.99, 130.76, 129.62, 128.61, 127.86, 127.61, 125.16, 123.02, 120.80, 119.43, 116.42, 115.67, 109.01, 67.32.

Compound 14c: m. p. 253.3–257.6^o^C. IR (KBr, cm^-1^): 3,219, 1,681, 1,558, 1,477, 1,285. HR-MS(ESI): Calcd. C23H16FN5O3 [M + H]^+^ *m/z*: 430.1315, found: 430.1319. ^1^H NMR (400MHz, DMSO-d_6_): 10.79 (s, 1H), 10.34 (s, 1H), 9.20 (s, 1H), 8.52 (s, 1H), 8.16 (d, J = 12.0Hz, 1H), 7.97–7.92 (m, 2H), 7.68–7.60 (m, 3H), 7.57 (d, J = 4.0Hz, 1H), 7.50 (t, J_1_ = 8.0Hz, J_2_ = 8.0Hz, 1H), 7.29–7.26 (m, 1H), 6.95 (d, J = 8.0Hz, 1H), 4.56 (s, 2H). ^13^C NMR (100MHz, DMSO-d_6_): 165.56, 165.52, 146.87, 139.97, 136.32, 134.29, 131.93, 130.72, 129.61, 128.80, 127.91, 127.61, 126.51, 126.11, 125.27, 125.16, 123.83, 117.80, 117.61, 116.41, 115.69, 109.03, 67.32.

Compound 14d: m. p. 244.6–251.4^o^C. IR (KBr, cm^-1^): 3,267, 1,693, 1,593, 1,494, 693. HR-MS(ESI): Calcd. C23H16ClN5O3 [M + H]^+^ *m/z*: 446.1020, found: 446.1096. ^1^H NMR (400MHz, DMSO-d_6_): 10.79 (s, 1H), 10.35 (s, 1H), 9.51 (s, 1H), 8.49 (s, 1H), 8.14 (d, J = 8.0Hz, 1H), 7.98 (dd, J_1_ = 8.0Hz, J_2_ = 8.0Hz, 2H), 7.70–7.65 (m, 2H), 7.61 (dd, J_1_ = 4.0Hz, J_2_ = 4.0Hz, 1H), 7.56 (s, 1H), 7.29–7.26 (m, 1H), 6.95 (d, J = 8.0Hz, 1H), 4.56 (s, 2H). ^13^C NMR (100MHz, DMSO-d_6_): 165.56, 147.37, 139.97, 138.12, 136.38, 134.77, 134.40, 132.21, 130.75, 129.62, 129.07, 128.62, 127.86, 127.61, 125.16, 120.81, 120.28, 119.05, 116.42, 115.67, 109.01, 67.32.

Compound 14e: m. p. 136.1–142.5^o^C. IR (KBr, cm^-1^): 3,409, 1,694, 1,589, 1,495, 757. HR-MS(ESI): Calcd. C23H16ClN5O3 [M + H]^+^ *m/z*: 446.1020, found: 446.1475. ^1^H NMR (400MHz, DMSO-d_6_): 10.81 (s, 1H), 10.37 (s, 1H), 9.16 (s, 1H), 8.52 (s, 1H), 8.14 (d, J = 8.0Hz, 1H), 7.95 (dd, J_1_ = 8.0Hz, J_2_ = 8.0Hz, 2H), 7.84–7.79 (m, 2H), 7.71–7.62 (m, 3H), 7.57 (s, 1H), 7.29–7.26 (m, 1H), 6.95 (d, J = 8.0Hz, 1H), 4.56 (s, 2H). ^13^C NMR (100MHz, DMSO-d_6_): 165.58, 165.53, 146.45, 139.94, 136.33, 134.94, 134.30, 132.35, 131.11, 130.82, 129.62, 129.09, 129.04, 128.93, 128.74, 127.86, 127.61, 125.10, 124.60, 116.43, 115.65, 108.98, 67.31.

Compound 14f: m. p. 261.1–265.4^o^C. IR (KBr, cm^-1^): 3,302, 1,689, 1,592, 1,497, 1,216, 516. ^1^H NMR (400MHz, DMSO-d_6_): 10.82 (s, 1H), 10.36 (s, 1H), 9.21 (s, 1H), 8.51 (s, 1H), 8.16 (d, J = 8.0Hz, 1H), 8.03 (d, J = 12.0Hz, 1H), 7.96–7.90 (m, 2H), 7.74–7.64 (m, 2H), 7.56 (s, 1H), 7.28 (d, J = 8.0Hz, 1H), 6.95 (d, J = 8.0Hz, 1H),4.56 (s, 2H). ^13^C NMR (100 MHz, DMSO) δ 165.58, 165.48, 155.38, 152.84, 146.98, 139.94, 136.31, 134.28, 130.56, 129.63, 129.33, 129.30, 128.80, 127.98, 127.69, 127.61, 125.17, 124.75, 124.64, 123.73, 123.69, 123.30, 123.21, 121.30, 121.07, 116.43, 115.64, 108.98, 67.30.

Compound 14g: m. p. 234.3–228.1^o^C. IR (KBr, cm^-1^): 3,302, 1,681, 1,563, 1,501, 1,217, 746. ^1^H NMR (400MHz, DMSO-d_6_): 10.83 (s, 1H), 10.38 (s, 1H), 9.25 (s, 1H), 8.52 (s, 1H), 8.17–7.95 (m, 3H), 7.72–7.57 (m, 4H), 7.30–7.28 (m, 1H), 6.96 (d, J = 8.0Hz, 1H),4.57 (s, 2H). ^13^C NMR (100 MHz, DMSO) δ 165.58, 154.39, 151.89, 146.94, 139.95, 136.33, 134.29, 130.53, 129.63, 129.60, 128.80, 128.01, 127.62, 125.95, 125.19, 119.68, 119.47, 116.43, 115.66, 108.99, 67.31.

### 5.2 Biological study

#### 5.2.1 Cell culture

Human cancer cell lines A549, Huh7, MCF-7, HCT-116, and SKOV3 were obtained from ATCC. The cells were cultured in DMEM or RPMI 1640 medium supplemented with 10% FBS and 1% penicillin/streptomycin and incubated at 37°C in a CO_2_-humidified atmosphere.

#### 5.2.2 CCK-8 assay and IC_50_ measurement

Cells were cultured in DMEM or RPMI 1640 medium containing 10% FBS and 1% penicillin/streptomycin at 37°C and 5% CO_2_. The CCK-8 assay was performed on several human cancer cell lines, including A549, Huh7, MCF-7, HCT-116, and SKOV3, according to the manufacturer’s instructions. Cells were plated at a density of 5 × 10^3^ cells per well in a 96-well plate. After 24 h of incubation, cells were treated with 40 μM compounds for 48 h. Cell viability was assessed by adding 10 μL of CCK-8 reagent, dissolved in the cell culture medium, and incubating for 1 h. Absorbance was measured at 450 nm using a microplate reader. IC_50_ values were determined using a dose-response curve with six concentrations (0, 10, 20, 40, 80, and 160 μM), and IC_50_ values were calculated using GraphPad Prism software.

#### 5.2.3 Live/dead viability assay

The live/dead viability assay was performed using the Calcein/PI Live/Dead Viability Assay Kit. A549 cells were plated at a density of 5 × 10^3^ cells per well in a 96-well plate and allowed to adhere overnight. Compounds were then added at concentrations of 0, 5, 10, or 20 μM for 24 h. After replacing the medium with fresh culture medium containing Calcein AM and propidium iodide, the cells were incubated for 30 min at 37°C in the dark. Live and dead cells were observed and photographed using a confocal microscope.

#### 5.2.4 Apoptosis quantification

A549 cells were plated at a density of 3 × 10^5^ cells per well in a six-well plate. Compounds at concentrations of 0, 5, 10, or 20 μM were added to the cells for 48 h. Cells were then collected, washed with PBS, and stained with Annexin V-FITC and propidium iodide for 20 min. Apoptotic cells were quantified using a flow cytometer, and the data were further analyzed using FlowJo software.

#### 5.2.5 Cellular RT-PCR level measurement

A549 cells were seeded in 12-well plates and treated with compounds for 48 h. Total RNA was extracted using TRIzol reagent, and RT-PCR was performed using PowerUp™ SYBR™ Green Master Mix. Gene expression was normalized to GAPDH. Primers were obtained from Aiji Biotech (Guangzhou, China) and are listed in Table 3. The results are expressed as mean ± SEM, and statistical significance was assessed using a two-tailed Student’s t-test (P < 0.05).

**TABLE 3:** List of oligonucleotide primer pairs used in RT-PCR analysis.

Gene	Forward primer (5′–3′)	Reverse primer (5′–3′)
P53	CCT​CAG​CAT​CTT​ATC​CGA​GTG​G	TGG​ATG​GTG​GTA​CAG​TCA​GAG​C
P21	AGG​TGG​ACC​TGG​AGA​CTC​TCA​G	TCC​TCT​TGG​AGA​AGA​TCA​GCC​G
H2AX	CGG​CAG​TGC​TGG​AGT​ACC​TCA	AGC​TCC​TCG​TCG​TTG​CGG​ATG
Bax	TCA​GGA​TGC​GTC​CAC​CAA​GAA​G	TGT​GTC​CAC​GGC​GGC​AAT​CAT​C
Bcl2	ATC​GCC​CTG​TGG​ATG​ACT​GAG​T	GCC​AGG​AGA​AAT​CAA​ACA​GAG​GC
Caspase3	GGA​AGC​GAA​TCA​ATG​GAC​TCT​GG	GCA​TCG​ACA​TCT​GTA​CCA​GAC​C
Caspase6	AGG​TGG​ATG​CAG​CCT​CCG​TTT​A	ATG​AGC​CGT​TCA​CAG​TTT​CCC​G
Caspase7	CGG​AAC​AGA​CAA​AGA​TGC​CGA​G	AGG​CGG​CAT​TTG​TAT​GGT​CCT​C
Keap1	CAA​CTT​CGC​TGA​GCA​GAT​TGG​C	TGA​TGA​GGG​TCA​CCA​GTT​GGC​A
Nrf2	AGG​TTG​CCC​ACA​TTC​CCA​AA	ACG​TAG​CCG​AAG​AAA​CCT​CA
Gpx4	TTGGTCGGCTGGACGAGG	GGGACGCGCACATGGT
Tnfa	ACT​GAA​AGC​ATG​ATC​CGG​GAC​G	AGC​AGG​CAG​AAG​AGC​GTG​GTG​G
ATG5	GGA​TGG​GAT​TGC​AAA​ATG​ACA​GA	TCC​TAG​TGT​GTG​CAA​CTG​TCC
ATG7	GGC​CAA​TAA​GAT​GGG​TCT​GA	GCT​TTT​GTC​CAC​TGC​TCC​TC

#### 5.2.6 Cellular ROS level measurement

A549 cells were seeded at a density of 5 × 10^3^ cells per well in a 96-well plate and allowed to adhere overnight. Compounds were then added at concentrations of 0, 5, 10, and 20 μM for 24 h. After treatment, the medium was removed, and fresh medium without FBS but containing 10 μM DCFH-DA was added and incubated in the dark for 30 min. The cells were washed three times with FBS-free medium to remove excess probe. ROS levels were then measured and imaged using confocal microscopy.

#### 5.2.7 Western blot analysis

The following antibodies were used for Western blot analysis: LC3, γ-H2AX, PARP, cyclin D, cyclin E, β-Catenin, and β-actin. Cells were treated with compounds (0, 5, 10, and 20 μM) for 48 h, and total cell proteins were extracted using RIPA buffer containing a protease/phosphatase inhibitor cocktail. Protein expression levels were measured and analyzed by Western blotting.

#### 5.2.8 DNA damage staining

A549 cells were seeded at a density of 2 × 10^3^ cells per well in 96-well plates. Cells were treated with different concentrations (0, 5, 10, 20, and 30 μM) of the compounds for 24 h. DNA damage was assessed using a DNA damage detection kit. The cell nuclei were stained blue, while DNA-damaged cells were stained green. Cells were then observed and photographed using a fluorescence microscope.

#### 5.2.9 Autophagy staining assay

A549 cells were seeded at a density of 2 × 10^3^ cells per well in a 96-well plate and treated with compounds at concentrations of 0, 5, 10, 20, and 30 μM for 24 h. After treatment, the medium was replaced, and cells were stained with monodansylcadaverine for 1 h in the dark at 37°C. Cells were then imaged using a confocal microscope.

#### 5.2.10 Molecular docking methods

The crystal structure of human DNA topoisomerase II beta (PDB ID: 4G0U; resolution: 2.70 Å) (Wu et al., 2013) was obtained from the RCSB Protein Data Bank (https://www.rcsb.org). The initial structure was prepared using the Protein Preparation Wizard in Maestro. Compounds 14b and 14c were prepared using the LigPrep module of the Schrödinger suite. The processed ligand structures were optimized to their three-dimensional forms with proper chirality and low energy using the OPLS4 force field. The docking area was defined by a grid box automatically generated by the Receptor Grid Generation tool based on the ligand position in the DNA of human topoisomerase II beta. The grid file was then used to dock the ligands to the DNA to identify potential binding modes.

#### 5.2.11 Statistical analyses

Data are presented as mean ± SEM and analyzed using Graph Prism 7.0. A two-tailed Student’s t-test or one-way analysis of variance followed by a Student–Newman–Keuls (SNK) test was used to assess significant differences. *P* < 0.05 was considered statistically significant.

## Data Availability

The original contributions presented in the study are included in the article/supplementary material; further inquiries can be directed to the corresponding authors.
